# Structural Basis of Human Dimeric α-Amino-β-Carboxymuconate-ε-Semialdehyde Decarboxylase Inhibition With TES-1025

**DOI:** 10.3389/fmolb.2022.834700

**Published:** 2022-04-07

**Authors:** Michele Cianci, Nicola Giacchè, Lucia Cialabrini, Andrea Carotti, Paride Liscio, Emiliano Rosatelli, Francesca De Franco, Massimiliano Gasparrini, Janet Robertson, Adolfo Amici, Nadia Raffaelli, Roberto Pellicciari

**Affiliations:** ^1^ Biochemistry and Structural Biology Laboratory, Department of Agricultural, Food and Environmental Sciences, Polytechnic University of Marche, Ancona, Italy; ^2^ TES Pharma S.r.l, Perugia, Italy; ^3^ Department of Pharmaceutical Sciences, University of Perugia, Perugia, Italy; ^4^ Department of Clinical Sciences DISCO, Section of Biochemistry, Polytechnic University of Marche, Ancona, Italy

**Keywords:** ACMSD, X-ray crystallography, TES-1025, decarboxylase, drug discovery, *de novo* NAD+ synthesis

## Abstract

Human α-amino-β-carboxymuconate-ε-semialdehyde decarboxylase (ACMSD) stands at a branch point of the *de novo* NAD^+^ synthesis pathway and plays an important role in maintaining NAD^+^ homeostasis. It has been recently identified as a novel therapeutic target for a wide range of diseases, including inflammatory, metabolic disorders, and aging. So far, in absence of potent and selective enzyme inhibitors, only a crystal structure of the complex of human dimeric ACMSD with pseudo-substrate dipicolinic acid has been resolved. In this study, we report the crystal structure of the complex of human dimeric ACMSD with TES-1025, the first nanomolar inhibitor of this target, which shows a binding conformation different from the previously published predicted binding mode obtained by docking experiments. The inhibitor has a *K*_*i*_ value of 0.85 ± 0.22 nM and binds in the catalytic site, interacting with the Zn^2+^ metal ion and with residues belonging to both chains of the dimer. The results provide new structural information about the mechanism of inhibition exerted by a novel class of compounds on the ACMSD enzyme, a novel therapeutic target for liver and kidney diseases.

## Introduction

Human α-amino-β-carboxymuconate-ε-semialdehyde decarboxylase (ACMSD, EC 4.1.1.45) (Fukuoka et al., 2002) stands at a branch point of the *de novo* NAD^+^ synthesis pathway, starting from the essential amino acid tryptophan, and plays an important role in maintaining NAD^+^ homeostasis. Given the beneficial effects of replenished NAD^+^ pools, there is an intense search for strategies to increase intracellular NAD^+^ by limiting NAD^+^ consumption or increasing NAD^+^ production (Katsyuba and Auwerx, 2017; Katsyuba et al., 2020). In this view, ACMSD inhibition is emerging as a potent strategy to replenish NAD^+^ levels by improving the coenzyme’s production (Yoshino, 2019).

**FIGURE 1:**
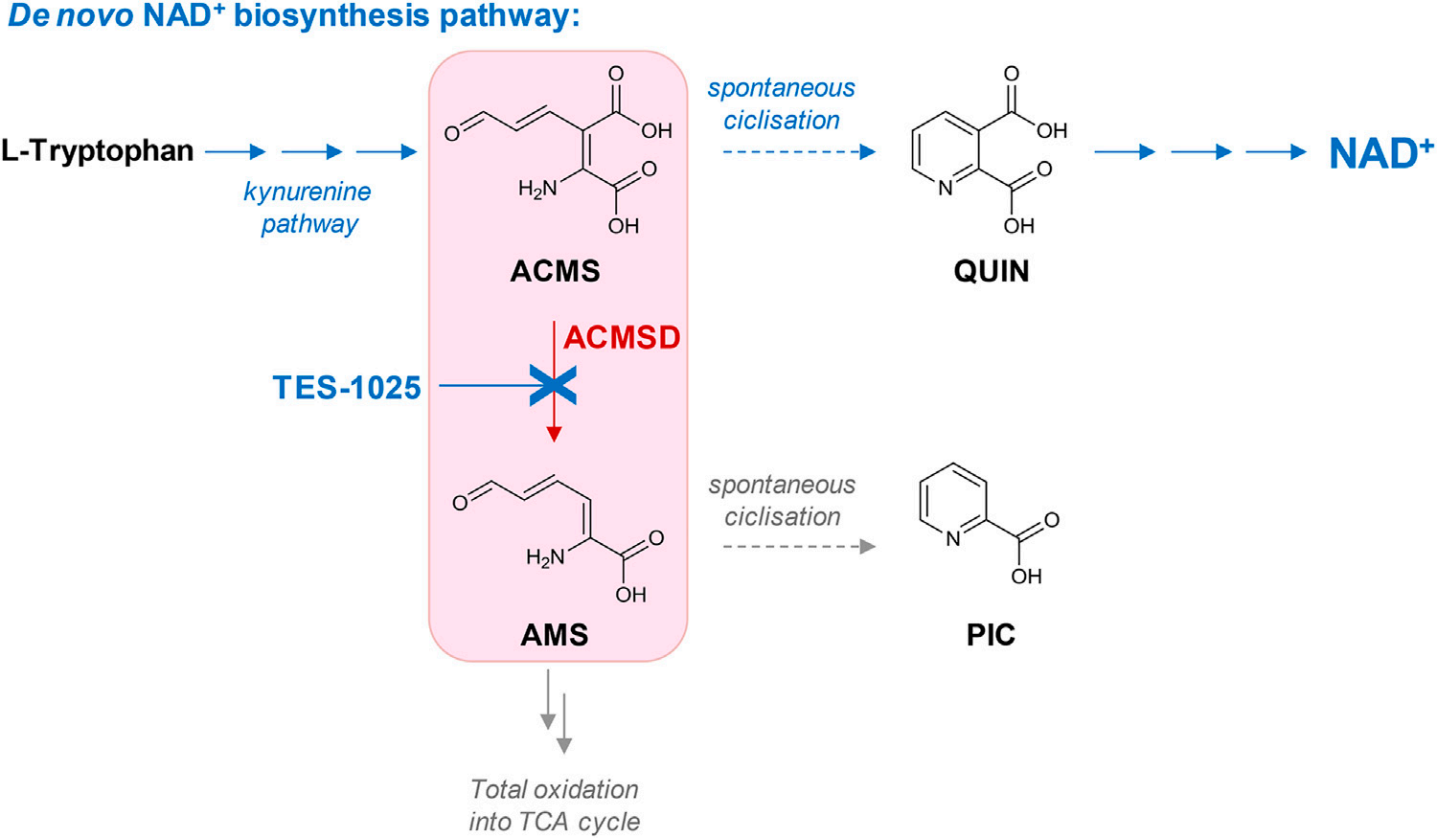
Role of ACMSD as the branching point in the kynurenine pathway leading to the *de novo* NAD^+^ biosynthesis. ACMSD catalyzes the decarboxylation of the ACMS intermediate to AMS metabolite toward total oxidation in the tricarboxylic acid (TCA) cycle. Inhibition by TES-1025 favors the flux from tryptophan through ACMS toward quinolinic acid (QUIN) and increases NAD^+^ production.

In detail, ACMSD catalyzes the decarboxylation of 2-amino 3-carboxymuconate 6-semialdehyde (ACMS), an intermediate in the *de novo* NAD^+^ synthesis pathway, to 2-aminomuconate-6-semialdehyde (AMS), through a metal-mediated, O_2_-independent, non-oxidative decarboxylation reaction (Li et al., 2006), which proceeds through a metal-bound hydroxide (Huo et al., 2013, 2015). AMS can either undergo spontaneous cyclization of the pyridine ring to form picolinic acid (PIC) or be oxidized to 2-aminomuconate, which is further metabolized so that it can enter the tricarboxylic acid (TCA) cycle. Otherwise ACMS, if not metabolized by ACMSD, can cyclize spontaneously to quinolinic acid (QUIN), which is further converted to the coenzyme NAD^+^ (Figure 1). Because the cyclization of ACMS into QUIN is a spontaneous reaction, the amount of ACMS undergoing this conversion and therefore leading to the production of NAD^+^ is primarily determined by the activity of ACMSD (Fukuoka et al., 2002). Thus, inhibition of ACMSD, which is primarily and highly expressed in the liver and kidneys (Pucci et al., 2007), would channel ACMS toward *de novo* NAD^+^ biosynthesis, providing a novel way to replenish NAD^+^ levels and re-establish NAD^+^ homeostasis in pathological conditions, particularly in liver- and kidney-associated diseases.

ACMSD is catalytically inactive in the monomeric form and active in the homodimeric form since the neighboring subunit contributes with one of the two substrate-binding arginine residues (Huo et al., 2013). Furthermore, recent experiments using size-exclusion chromatography coupled with small-angle X-ray scattering (SEC-SAXS) analysis have evidenced a protein concentration-dependent activity of the enzyme, revealing that its quaternary structure is in a dynamic equilibrium among the monomeric, dimeric, and higher-order oligomeric states (Yang et al., 2019).

**FIGURE 2:**
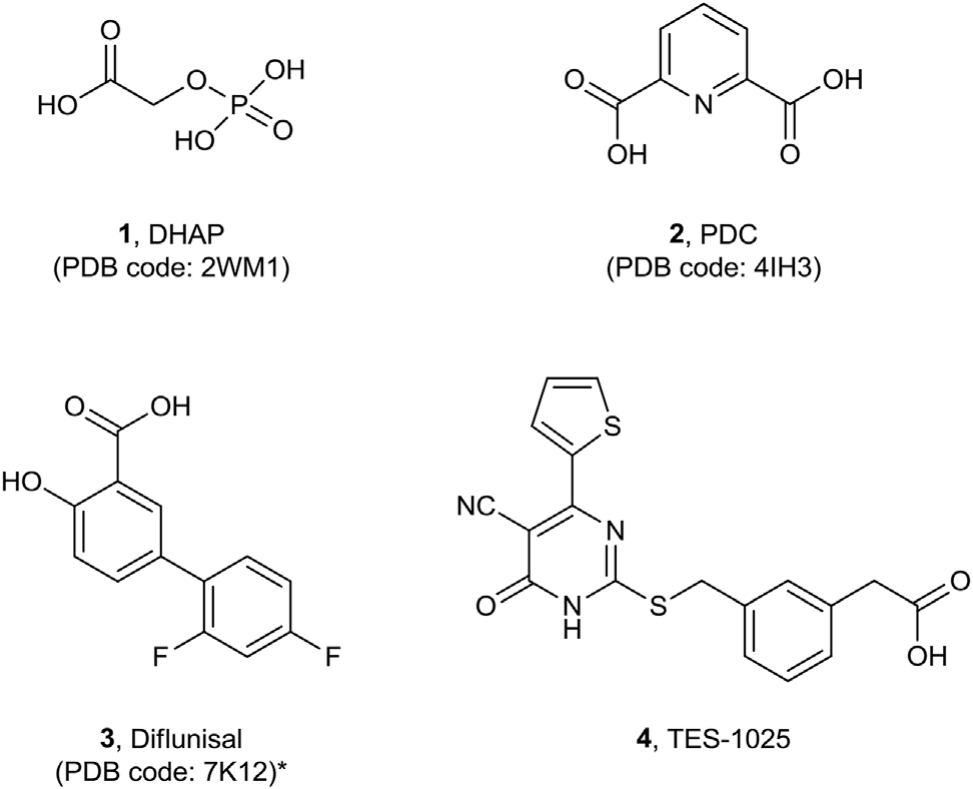
Molecular structures of published co-crystalized ACMSD inhibitors.*: diflunisal has been resolved in *P. fluorescens* ACMSD.

The first small molecule inhibitors of ACMSD to be identified were the anti-tuberculosis drug pyrazinamide (Saito et al., 2000) and the phthalate monoester, such as mono (2-ethylhexyl)phthalate (MEHP) (Fukuwatari et al., 2004) with weak and nonselective activity. Subsequent efforts in understanding the mechanism of recognition and binding of ligands to the active site of the human enzyme resulted in the release of the first co-crystal complex of ACMSD (PDB code 2WM1) with the inhibitor 1,3-dihydroxyacetonephosphate (DHAP, **1**) (Garavaglia et al., 2009) (Figure 2).

Subsequently, the structure of the human recombinant ACMSD complex with the competitive inhibitor pyridine-2,6-dicarboxylic acid (PDC, **2**) (PDB code 4IH3) was published, refining the previous findings (Huo et al., 2015) (Figure 2).

Recently, the salycilic-derivative, nonsteroidal anti-inflammatory drug (NSAID) and FDA-approved drug diflunisal (**3**) was identified to inhibit ACMSD with an IC_50_ of 13.5 µM, and its complex structure in the *Pseudomonas fluorescens* ACMSD has been resolved and published (PDB code 7K12) (Yang et al., 2021) (Figure 2). A chronological summary of all ACMSD structures that have been resolved and published is reported in Table 1.

**TABLE 1:** Summary of all ACMSD X-ray structures deposited in the Protein Data Bank (PDB) (order for date of release).

PDB code	Date of release	Resolution (Å)	Organism	Conformation	Mutation	Metal cofactor	Ligand	References
2HBX	2006-09-19	2.50	*Pseudomonas fluorescens*	Dimer	No	Co^2+^	—	Martynowski et al. (2006)
2HBV	2006-09-19	1.65	*Pseudomonas fluorescens*	Dimer	No	Mg^2+^, Zn^2+^	—	Martynowski et al. (2006)
2WM1	2009-11-03	2.01	*Homo sapiens*	Monomer (but dimer in the lattice)	No	Zn^2+^	DHAP	Garavaglia et al. (2009)
4EPK	2012-08-22	2.60	*Pseudomonas fluorescens*	Dimer	H228G	Mg^2+^, Zn^2+^	—	Huo et al. (2012)
4ERI	2012-08-22	2.00	*Pseudomonas fluorescens*	Dimer	H228Y	Mg^2+^, Zn^2+^	—	Huo et al. (2012)
4ERA	2012-08-22	2.40	*Pseudomonas fluorescens*	Dimer	H228Y	Co^2+^	—	Huo et al. (2012)
4ERG	2012-08-22	2.79	*Pseudomonas fluorescens*	Dimer	—	Fe^3+^	—	Huo et al. (2012)
4IGM	2014-05-07	2.39	*Homo sapiens*	Dimer	No	Zn^2+^	—	No references
4IGN	2014-05-07	2.33	*Homo sapiens*	Dimer	R47A	Zn^2+^	—	Huo et al. (2015)
4IH3	2014-05-21	2.49	*Homo sapiens*	Dimer	No	Zn^2+^	PDC	Huo et al. (2015)
4OFC	2014-11-19	1.99	*Homo sapiens*	Dimer	No	Zn^2+^	—	Huo et al. (2015)
6MGS	2019-06-19	3.13	*Pseudomonas fluorescens*	Dimer	No	Co^2+^	—	Yang et al. (2019)
6MGT	2019-06-19	2.77	*Pseudomonas fluorescens*	Dimer	H110A	Co^2+^	—	Yang et al. (2019)
7K12	2021-01-13	2.17	*Pseudomonas fluorescens*	Dimer	No	Zn^2+^	Diflunisal	Yang et al. (2021)
7K13	2021-01-13	1.83	*Pseudomonas fluorescens*	Dimer	No	Zn^2+^	Diflunisal-derivative	Yang et al. (2021)

TES-1025 (CAS. 1883602-21-8, 2-[3-[(5-cyano-6-oxo-4-thiophen-2-yl-1H-pyrimidin-2-yl) sulfanylmethyl]phenyl]acetic acid, PubChem CID: 137142885) is a potent and selective human α-amino-β-carboxymuconate-ε-semialdehyde decarboxylase (ACMSD) inhibitor with an IC_50_ of 13 nM. The compound has been selected as the first low-nanomolar inhibitor of human ACMSD with a suitable overall balance of good physicochemical properties and *in vitro* safety profile, identified after the discovery, synthesis, and biological evaluation of a series of 2-thiopyrimidone-5-carbonitriles as the first class of small-molecule drug-like ACMSD inhibitors. Proof-of-concept studies for the first time revealed that the inhibition of ACMSD by TES-1025 led to the modulation of intracellular NAD^+^ levels with consequent *in vivo* enhancement of *de novo* NAD^+^ biosynthesis via ACMSD target engagement (Pellicciari et al., 2018).

On the basis of the discovery of TES-1025 and related analogs, we have established valuable tools for a better understanding of the therapeutic applications of ACMSD inhibitors for disorders such as mitochondrial dysfunctions and metabolic and renal diseases, associated with the dysregulation or reduced NAD^+^ levels. *In vivo* efficacy data obtained with TES-1025 in preclinical murine models of liver and kidney diseases (Katsyuba et al., 2018) suggested ACMSD as a promising novel therapeutic target to improve health in pathological settings such as that of acute kidney injury (AKI) (Kellum and Prowle, 2018; Manrique-Caballero et al., 2021).

In this study, we report the crystal structure of the complex of human dimeric ACMSD with TES-1025 (**4**), (Figure 2) the first potent and selective ACMSD inhibitor.

## Materials and Methods

### Expression and Purification of hACMSD

Expression of the recombinant protein was achieved as described previously (Pucci et al., 2007). Purification was performed as described previously (Garavaglia et al., 2009), with some modifications. In detail, a pellet of *Pichia pastoris* cells expressing the enzyme and derived from 400 ml culture was resuspended in 80 ml of lysis buffer consisting of 10 mM potassium phosphate, pH 7.0, 50 mM NaCl, 1 mM DTT, 5 mM 2-mercaptoethanol, and 1 mM PMSF and aprotinin, leupeptin, chymostatin, pepstatin, and antipain at 0.002 mg/ml each. After disruption by two cycles of French press (SML-Aminco, Urbana, IL, United States) at 1,000 psi, the suspension was centrifuged at 40,000 × g, for 30 min at 4°C. The supernatant was made to 10 mg/ml by dilution with lysis buffer, and streptomycin sulfate was added dropwise at a final concentration of 1%. After 30 min stirring on ice, the sample was centrifuged at 20,000 × g for 10 min at 4°C, and the supernatant was applied to 4 ml TALON Superflow resin (Cytiva, United States) equilibrated with 50 mM potassium phosphate pH 7.4 and 50 mM NaCl (buffer A). After washing with 20 mM imidazole in buffer A, elution was performed with 350 mM imidazole in buffer A.

### Kinetics Studies

The inhibitory effect of TES-1025 on ACMSD activity was determined by the coupled spectrophotometric assay (Pucci et al., 2007). Briefly, pre-assay mixtures consisting of different concentrations of hydroxyanthranilic acid (from 5 to 20 µM) and an excess amount of recombinant *R. metallidurans* hydroxyanthranilic acid dioxygenase, in 50 mM 4-morpholinepropanesulfonic acid, pH 6.5 and 100 mM ammonium iron sulfate, were incubated at 37°C, with monitoring ACMS formation at 360 nm. After the reaction was complete, 30 nM ACMSD and TES-1025 (from 0.5 to 40 nM) were added. The enzyme activity was calculated by the initial rate of the absorbance decrease subtracted from that of a control mixture in the absence of ACMSD. The *K*_*i*_ value was calculated from the initial velocity data using the Dixon equation for tightly bound competitive inhibitor (Segel, 1993):

$$\left[I_{t}\right]=\left(K_{i}\left(1+\frac{\left[S\right]}{K_{m}}\right)\frac{V_{0}}{V_{i}}+\left[E_{t}\right]\right)\;*\left(1-\frac{V_{i}}{V_{0}}\right),$$ (1)

where *V*_*i*_ is the initial velocity at a given [*S*] in the presence of the inhibitor, *V*_*o*_ is the initial velocity at the same [*S*] in the absence of the inhibitor, *K*_*m*_ is the Michaelis–Menten constant for the substrate, [*I*_*t*_] and [*E*_*t*_] are the total amount of the inhibitor and enzyme, respectively, and *K*_*i*_ is the apparent *K*_*i*_ value. The intercept on the *y*-axis of the replot of apparent *K*_*i*_ values against [*S*] gives the *K*_*i*_ value.

### Protein Crystallization and Data Collection

TES-1025 was developed by TES Pharma as reported by Pellicciari et al., (2018). For the crystallization trials, the TALON pool containing the purified protein was diluted ten-fold with 50 mM potassium phosphate and 5 mM 2-mercaptoethanol and then concentrated by ultrafiltration, using an Amicon Ultra Centrifugal Filter (cutoff 10 kDa, Merck, Millipore), at 4°C, to a final protein concentration of 4.5 mg/ml. For all crystallization trials, the sitting drop vapor diffusion method was applied. The concentrated enzyme was incubated at the 1:10 molar ratio with a stock solution of 50 mM TES-1025 in dimethyl sulfoxide, for 1 hour at room temperature, and 1.25 μL of protein–ligand solution was mixed with an equal volume of reservoir solution, and it was equilibrated against 100 µL of the reservoir solution. The best crystals were obtained in reservoir solution containing 100 mM Na(CH_3_COO), pH 5.7, 22% (w/v) PEG 4000, and they grew to their final size in few weeks at 18°C.

Crystals were transported to the synchrotron in plates, mounted in nylon loops, and flash-frozen directly at 100 K in a nitrogen gas stream. Diffraction data were collected at the European Synchrotron Radiation Facility (ESRF, Grenoble, France) at beamline ID30A-3 (MASSIF-3) (von Stetten et al., 2020).

### Structure Determination, Refinement, and Analysis

The diffraction data were integrated and scaled with the XDS/XSCALE program package (Kabsch, 2010). The crystals belong to the space group P2_1_2_1_2, with unit cell a = 153.3 Å, b = 92.5 Å, and c = 103.9 Å. Starting phases for solving the crystal structure were obtained with molecular replacement using PHASER (Adams et al., 2002) with monomer A of the hACMSD structure as a starting model reported by Huo et al. (2015) (PDB code 4IH3) after atom randomization to avoid any bias as a search model. Automated model building was accomplished by the PHENIX (Adams et al., 2002) suite, followed by manual fitting of the side chains and solvent molecules into electron density maps performed using COOT (Emsley and Cowtan, 2004) and PHENIX suite (Adams et al., 2002), while monitoring R_work_, R_free_, and Ramachandran plot with PROCHECK (Laskowski et al., 1993) and related geometrical parameters. The Fourier difference electron density OMIT maps at 3σ were inspected to verify the presence of TES-1025. The models were checked with the PDB REDO web server (Joosten et al., 2014). Model coordinates and structure factors of the X-ray crystal structure of ACMSD co-crystallized in the presence of TES-1025 were deposited in the Protein Data Bank (PDB) under the accession code: 7PWY. Data collection, processing, and final refinement statistics are given in Table 2. Ligand interaction diagram is generated by the tool of Maestro of the Schrodinger suite 2017−1 (https://www.schrodinger.com). The images produced in this article were generated using CCP4mg (McNicholas et al., 2011) and PyMOL software (https://www.pymol.org).

**TABLE 2:** Data collection and refinement statistics.

Wavelength (Å)	0.967
Space group	P 2_1_2_1_2
Cell parameters (a, b, and c, Å)	153.4, 92.6, 103.9
Resolution range (Å)	45.89–2.50 (2.50–2.58)^a^
Total reflections	395,775 (30,276)^a^
Unique reflections	51,945 (4,423)^a^
Redundancy	7.6 (6.8)^a^
Completeness (%)	99.9 (100.0)^a^
Mean I/sigma(I)	11.5 (1.6)^a^
R_merge_ ^b^	0.11 (11.1)^a^
R_pim_ ^c^	0.063 (0.688)^a^
CC1/2	0.999 (0.766)^a^
CC*	1.00 (0.926)^a^
Reflections used in refinement	51,611 (5,113)^a^
Reflections used for R_free_	2,565 (292)^a^
Wilson B-factor (Å^2^)	52.35
R_work_ ^d^	0.210 (0.310)^a^
R_free_ ^d^	0.252 (0.358)^a^
Total no. of atoms^d^	10,647
Macromolecules	10,296
Ligands	62
Water molecules	289
Protein residues	1,289
RMSD^d^	—
Bond length (Å)	0.003
Angles (°)	0.55
Ramachandran^d^	—
Favored (%)	96.54
Allowed (%)	3.46
Outliers (%)	0.00
Average B-factor^d^	68.28
Macromolecules	68.48
Ligands	73.64
Solvent	59.83

a Values in the highest resolution shell.

b ${\text{R}}_{\text{merge}}=\underset{\text{hkl}}{\sum }\underset{\text{j}}{\sum }\left|{\text{I}}_{\text{j}}-\text{I}\right|/\underset{\text{hkl}}{\sum }\underset{\text{j}}{\sum }{\text{I}}_{\text{j}}$ , where I is the intensity of a reflection, and I is the mean intensity of all symmetry-related reflections j.

c ${\text{ R}}_{\text{p}.\text{i}.\text{m}.}=\underset{\text{hkl}}{\sum }\left\{{\left[1/\left(\text{N}-1\right)\right]}^{1/2}\underset{\text{j}}{\sum }\left|{\text{I}}_{\text{j}}-\text{I}\right|\right\}/\underset{\text{hkl}}{\sum }\underset{\text{j}}{\sum }{\text{I}}_{\text{j}}$ , where I is the intensity of a reflection, and I is the mean intensity of all symmetry-related reflections j, and N is the multiplicity (Weiss, 2001).

d Calculated with PHENIX suite (Adams et al., 2002), R_free_ is calculated using 5% of the total reflections that were randomly selected and excluded from refinement.

## Results

The human ACMSD crystal structure in complex with TES-1025 at 2.5 Å resolution was refined to final R_work_ and R_free_ values of 0.210 and 0.252, respectively. The Ramachandran plot shows more than 96% of the residues in the favored regions and 4% in the allowed regions (Table 2). The molecular replacement solution of the hACMSD structure in complex with TES-1025 comprised four monomers in the asymmetric unit to form two homodimers. The average RMSD, calculated with SUPERPOSE (Winn et al., 2011) over 320 residues of each monomer, against the starting model, is 0.45 Å, to confirm that the overall fold of the enzyme is maintained. In brief, hACMSD shows a molecular architecture comprising of 12 α-helices, 11 β-strands, and the connecting loops. Residues 14–48 form the small insertion domain that comprises a short α-helix and a three-stranded anti-parallel β-sheet; the remaining protein residues form a (α/β)_8_ barrel domain and a two-α-helices C-terminal extension.

**FIGURE 3:**
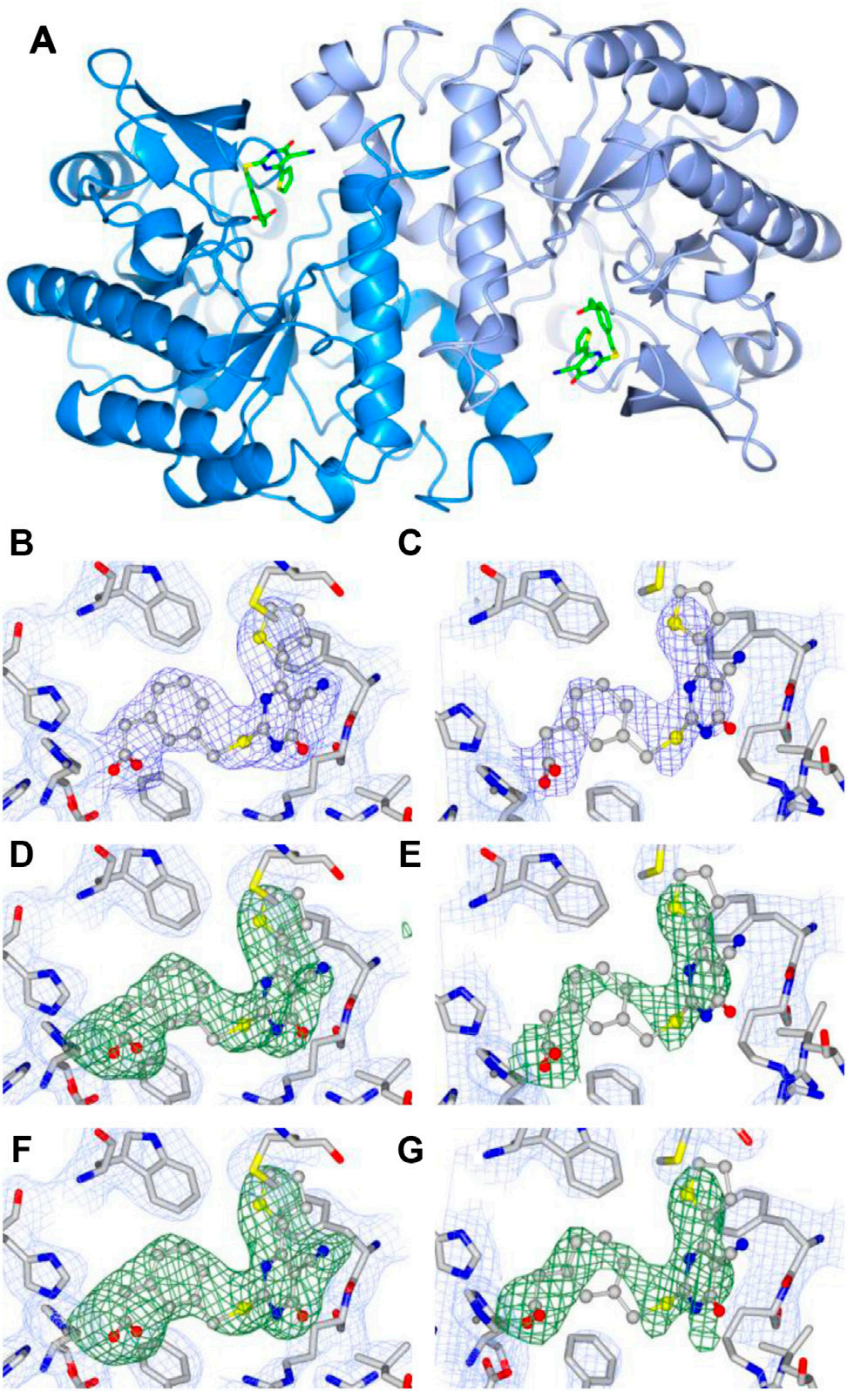
**A)** Ribbon diagram of the dimeric structure of human ACMSD in complex with TES-1025: dark blue, monomer A; light blue, monomer B. Fourier difference maps of TES-1025 molecules bound to monomers A and B, respectively: **(B,C)** 2F_o_-F_c_ map (light blue for the protein and blue for the ligand), contoured at 1σ level; **(D,E)** F_o_-F_c_ omit map (green for the ligand) contoured at 3σ level; (**F,G)** F_o_-F_c_ polder map (green for the ligand) contoured at 4σ level.

A total of two homodimers are observed in the asymmetric unit. Analysis of interface area, solvation energy gain upon interface formation, and the total binding energy of the interface, calculated using PISA (Krissinel and Henrick, 2007), confirms homodimers A, B and C, D as biological units. hACMSD homodimers have been reported previously (Garavaglia et al., 2009; Huo et al., 2015) (Figure 3A). In homodimer AB, there are no disordered regions, while in homodimer CD there are disordered regions namely in chain C between residues 30–37, 178–185, and 242–253 and in chain D between residues 242–253.

Similar to previously published X-ray structures (Garavaglia et al., 2009; Huo et al., 2015), the active site of hACMSD is located at the C-terminal opening of the β-barrel and characterized by a Zn^2+^ metal ion coordinated with a distorted trigonal bipyramid geometry, by residues His6, His8, His174, His 224, the moiety of Asp291, and a conserved water molecule. A water molecule bridges the Zn^2+^ metal ion with the guanidino moiety of Arg235 belonging to the neighboring subunit (chain B) of the hACMSD dimer.

The presence and positioning of the TES-1025 molecules was indicated by the Fourier difference 2F_o_-F_c_ maps at 1σ, the Fourier difference F_o_-F_c_ OMIT maps at 3σ level, and the polder maps at 4σ level in each active site of hACMSD homodimer AB (Figure 3). In homodimer CD, no electron density was observed that could be attributed to a TES-1025 molecule. The molecule TES-1025 located in monomer A was refined with full occupancy, and the molecule located in monomer B was refined with a partial occupancy of 0.80. The correlation coefficients for the polder map (Liebschner et al., 2017), calculated by omitting both TES-1025 molecules were CC (1, 3) = 0.89, i.e., larger than CC (1, 2) = 0.54 and CC (2, 3) = 0.56. When omitting only TES-1025 molecule in chain A, we obtained CC (1, 3) = 0.91, i.e., larger than CC (1, 2) = 0.62 and CC (2, 3) = 0.55. When omitting only TES-1025 molecule in chain B, we obtained CC (1, 3) = 0.84, i.e., larger than CC (1, 2) = 0.59 and CC (2,3) = 0.57. Since the CC (1, 3) is larger than CC (1, 2) and CC (2, 3), then the density observed corresponds to the atomic features of the TES-1025 molecules.

**FIGURE 4:**
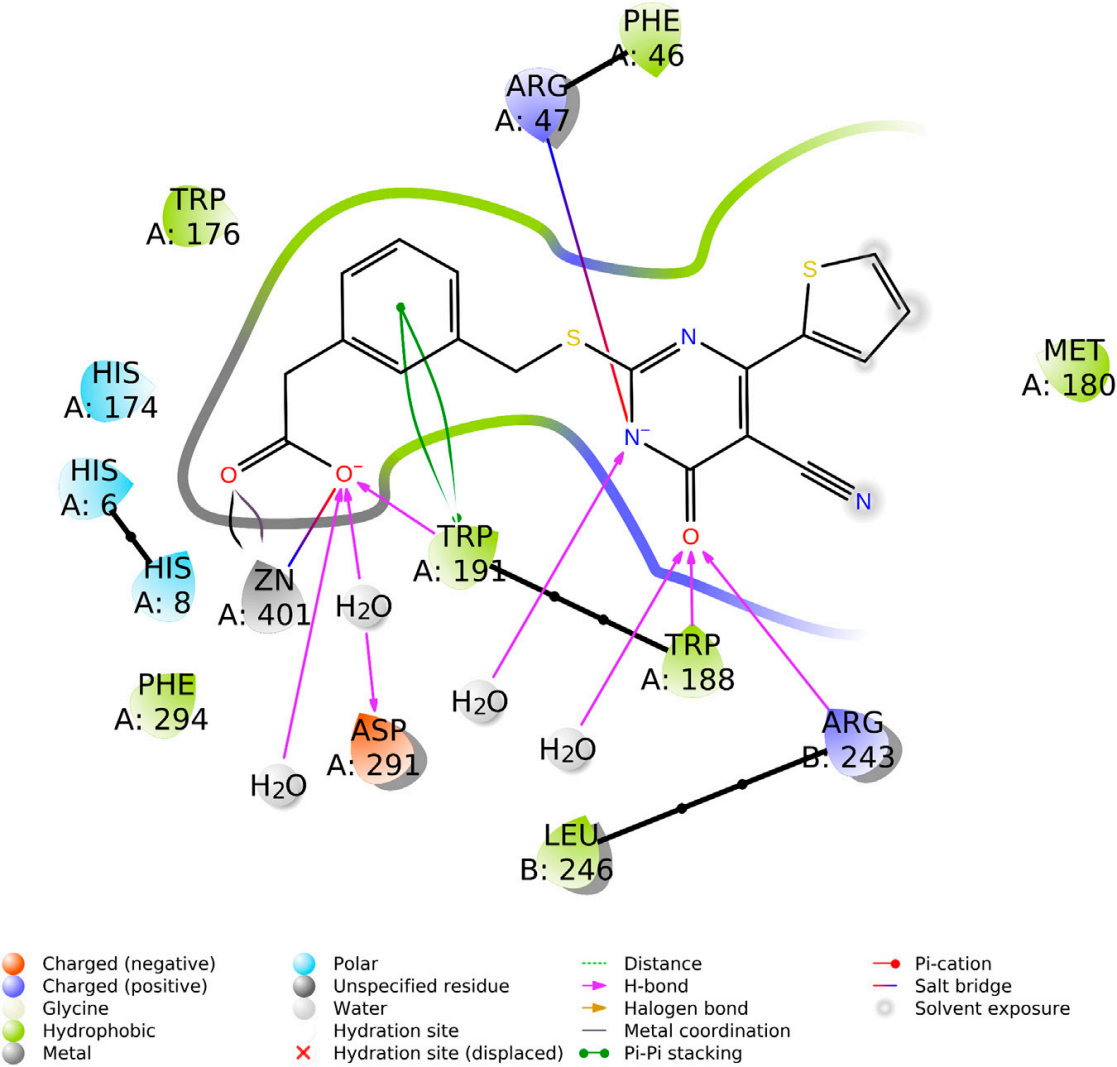
Ligand interaction diagram of TES-1025 in the ACMSD active site of monomer A, reporting the ligand–protein type of interactions and involved residues (diagram calculated and generated by Maestro, Schrodinger suite).

The active site of each subunit binds one TES-1025 molecule with the same set of interactions. The carboxylic moiety of the ligand coordinates the Zn^2+^metal ion (Zn···O distance is 2.5 Å) and establishes an H-bond with the indole moiety of Trp191 residue (the H···O distance is 2.6 Å, and the N-H···O angle is 135.8°) while interacts with Asp291 through a water molecule. The pyrimidine ring interacts through the carbonyl group with Arg243 (the H···O distance is 2.5 Å, and the N-H···O angle is 143.6°), belonging to the neighboring subunit (chain B) of the functional dimer of ACMSD, while a second H-bond is established with the catalytic residue Trp188 (the H···O distance is 2.7 Å, and the N-H···O angle is 132.5°). The negatively charged nitrogen of the TES-1025 pyrimidine ring engages a charge–charge interaction with the positively charged nitrogen of the Arg47 (the N···N distance is 3.6 Å). Also, further interactions with solvent water molecules are defined. The 2-thiophene ring fits into a hydrophobic cavity generated by Trp176, Phe46, Met180, and Trp191; this latter residue also makes Pi–Pi stacking with the phenyl ring of TES-1025. (Figure 4).

**FIGURE 5:**
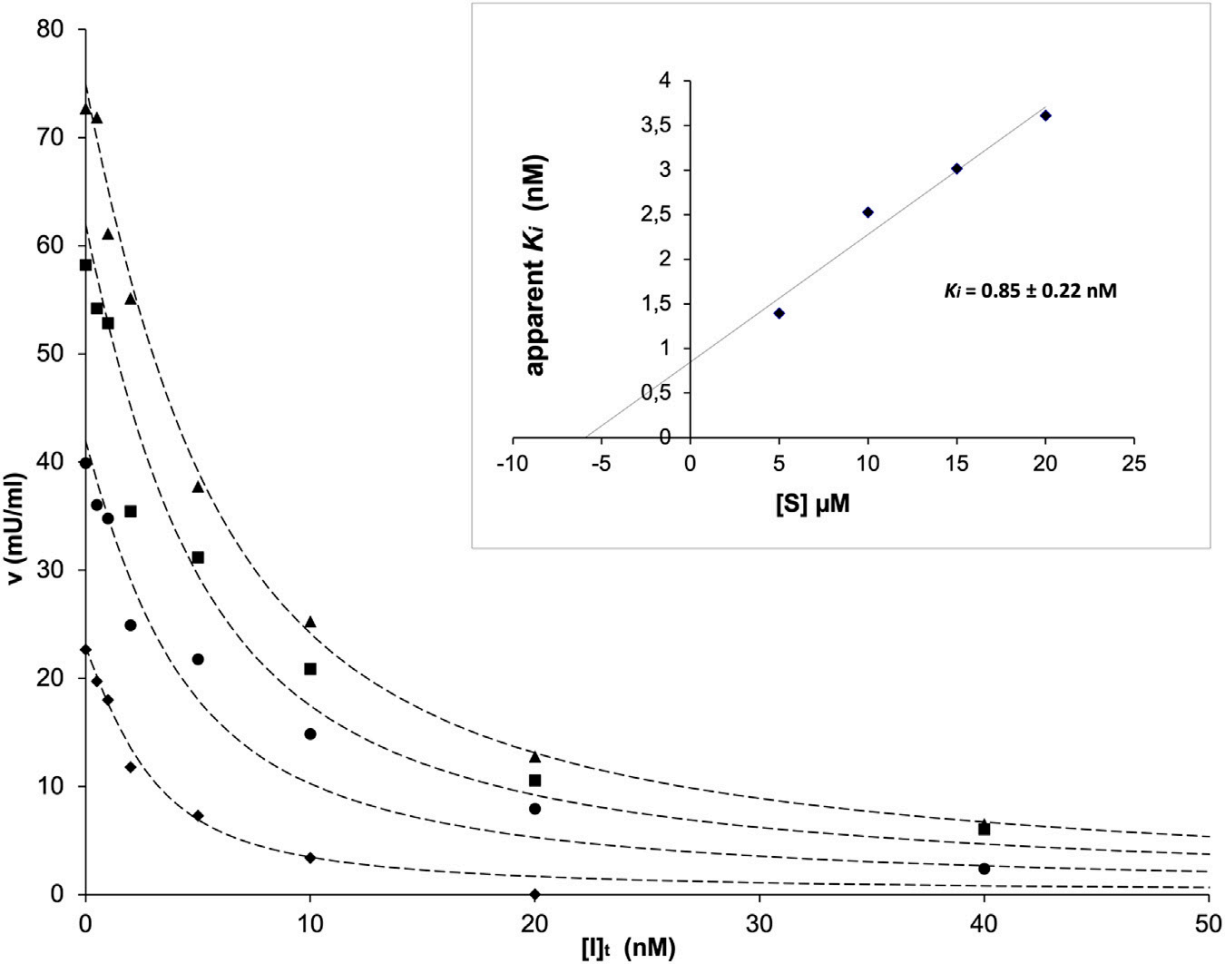
ACMSD activity as a function of inhibitor concentration in the presence of fixed concentrations of the substrate traced with diamonds (5 μM), circles (10 μM), squares (15 μM), and triangles (20 μM). In the inset, the replot of apparent *K*_*i*_ versus [*S*] is shown.

The kinetic analysis of the inhibition exerted by TES-1025 on ACMSD was performed by assaying the enzyme activity in the presence of varying concentrations of the inhibitor and substrate. The IC_50_ value of the inhibitor at 10 µM substrate concentration is reported to be about 13 nM (Pellicciari et al., 2018). This value is very close to the concentration of ACMSD which is used in the activity assay, implying that a significant portion of the total inhibitor in the assay mixture is enzyme-bound. Therefore, to investigate the inhibition kinetics, the Dixon method (Segel, 1993) was used as described in Materials and Methods. The best fit with the experimental data was obtained by using the Dixon Equation 1 for a tightly bound competitive inhibitor (Figure 5). A K_i_ value of 0.85 ± 0.22 nM was calculated (inset in Figure 5).

## Discussion

The structural basis of human dimeric α-amino-β-carboxymuconate-ε-semialdehyde decarboxylase inhibition with TES-1025 is severalfold. Overall, the X-ray structure of the ACMSD–TES-1025 complex described in this study experimentally confirmed the competitive inhibition mode displayed by the ligand, in which the catalytic Arg47 and the Zn^2+^ metal ion together with Trp191 and chain B are engaged in unique specific interactions with the meta carboxylate group and the pyrimidine ring of the ligand. These two interactions lock the head and the tail of the ligand, respectively. Moreover, the pyrimidine ring strongly interacts with the pocket residues side chains through a network of hydrogen bonds mediated by water molecules, thus stabilizing the central core of the ligand. This strong network of interactions generated by TES-1025 ensured the low nanomolar potency inhibition of the enzyme and confirmed the previously published data of structure and activity relationship (Pellicciari et al., 2018). The kinetic analysis confirmed that TES-1025 is a competitive inhibitor with a *Ki* value in the low nanomolar range.

**FIGURE 6:**
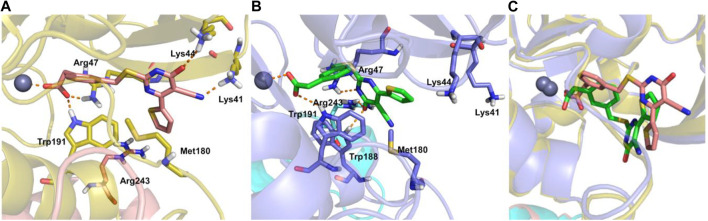
TES-1025-binding (pink sticks) modes in the docking model **(A)**, TES-1025 (green sticks) in the crystal structure **(B)**, and superposition of the two ligand poses **(C)**, together with the protein ribbons and Zn^2+^ ions. ACMSD chains A and B are shown in yellow and pink ribbons for the induced fit result, while are in blue and cyan ribbons for the X-ray image, respectively. The Zn^2+^ metal ion is displayed as a gray ball. The relevant residues of the binding site are labeled and shown as sticks. Hydrogen bond and salt bridge interactions are indicated with orange dashed lines.

In the article by Pellicciari et al. (2018), the investigation of the induced-fit docking pose of TES-1025 into the hACMSD catalytic site coming from the 4IH3 X-ray (Huo et al., 2015) was presented. The results indicated that the majority of the binding affinity was due to the ionic interaction between the m-carboxy group of the ligand with the Zn^2+^ metal ion and the catalytic residues Arg47 and Trp191. Meanwhile, the pyrimidine ring was strongly anchored to Lys41 and Lys44 residues by hydrogen bond and ionic interactions. Furthermore, additional hydrophobic contacts were also established between the thiophene and Met180. In this view, the current X-ray structure of ACMSD in complex with TES-1025 reports a different ligand disposition with respect to the putative binding mode predicted by docking computation (ligand RMSD calculated on non-hydrogen atoms is 6.42 Å when using the protein backbone for the alignment). Indeed, upon TES-1025 binding, the side chain of Arg47, as resolved in the crystal structure, is oriented to the middle of the pocket driven by the interaction with the acidic oxygen of the pyrimidine ring of the ligand. It is worth noting that this Arg47 orientation was observed neither in the 4IH3 complex used for the *in silico* studies nor in other released ACMSD X-ray structures (pdb codes: 2WM1, 4IGM, 4IGN, and 4OFC) (Garavaglia et al., 2009; Huo et al., 2015). Moreover, the carbonyl moiety of the pyrimidine ring of TES-1025 engages an H-bond with Arg243, belonging to the neighboring subunit (chain B) of the functional dimer of ACMSD. The crystallographic disposition showed a 180° rotation of the pyrimidine ring around the Csp^2^-S bond that completely abolishes the interactions present in the docking pose with Lys44 and Lys41 (Figure 6).

The previous detailed works of the Aimin Liu lab (Huo et al., 2013; Huo et al., 2015) confirmed the metal ion (Zn^2+^) dependence of the human ACMSD for its catalytic activity and the active role of Arg47 and Arg235 of the chain B for the interaction of the natural substrate ACMS in the homodimeric functional complex. TES-1025 was able to interact with all the essential catalytic features of the site, with only the substitution of Arg235 with Arg243, stabilizing a homodimeric inactive complex and confirming the high affinity and potency of the ligand. Moreover, the preferential recognition and binding of the pyrimidine moiety of TES-1025 in ACMSD supported the subfamilial similarities with the related enzyme 5-carboxyl-uracil decarboxylase (IDCase) (Huo et al., 2015).

The asymmetric unit consists of two homodimers, namely AB and CD. In AB, two TES-1025 molecules were observed, while in CD no electron density was observed that could be attributed to a TES-1025 molecule. The two homodimers display high dynamics of the different chains too. Indeed, in homodimer AB, there are no disordered loop regions, while in homodimer CD there are disordered regions. Considering that upon ligand binding, Arg243 interacts with the pyrimidine ring of TES-1025, and the presence of the ligand is reflected in the folding of the residue region 240–253 to an α-helix structure.

In summary, the determination of the crystal structure of the human ACMSD homodimer with the first nanomolar and selective inhibitor TES-1025 reveals unforeseen interactions of the functional groups of the small molecule with the catalytic side chains and the metal ion within the active site, elucidating the principles of its potent inhibitory mechanism. These results further validate the selectivity of TES-1025 for the enzyme and consolidate the knowledge about ACMSD as a promising therapeutic target (Katsyuba et al., 2018; Kellum and Prowle, 2018; Kellum et al., 2020; Manrique-Caballero et al., 2021) for the recovery of the *de novo* NAD^+^ biosynthesis pathway and the maintaining of NAD^+^ homeostasis impaired in hepatic (Zhou et al., 2016) and renal diseases (Poyan Mehr et al., 2018).

## Data Availability

Structure factors and coordinates have been deposited in the Protein Data Bank with the PDB code: 7PWY.
